# Supplementary material for: Age and Task Modulate Olfactory Sensitivity in the Florida Carpenter Ant Camponotus floridanus
Source: Insects. 2023 Aug 23;14(9):724. doi: 10.3390/insects14090724 (PMC10532128; doi:10.3390/insects14090724)
Supplement: Supplementary file 1 [file insects-14-00724-s001.zip › insects-2537344-supplementary.pdf]

insects

Article

Age and Task Modulate Olfactory Sensitivity in the Florida Carpenter Ant *Camponotus floridanus*

Stephen T. Ferguson, Isaac Bakis, Nicholas D. Edwards and Laurence J. Zwiebel *

Department of Biological Sciences, Vanderbilt University, Nashville, TN 37235, USA; stephen.t.ferguson@vanderbilt.edu (S.T.F.); isaac.bakis@vanderbilt.edu (I.B.); nicholas.d.edwards@vanderbilt.edu (N.D.E.)
* Correspondence: l.zwiebel@vanderbilt.edu

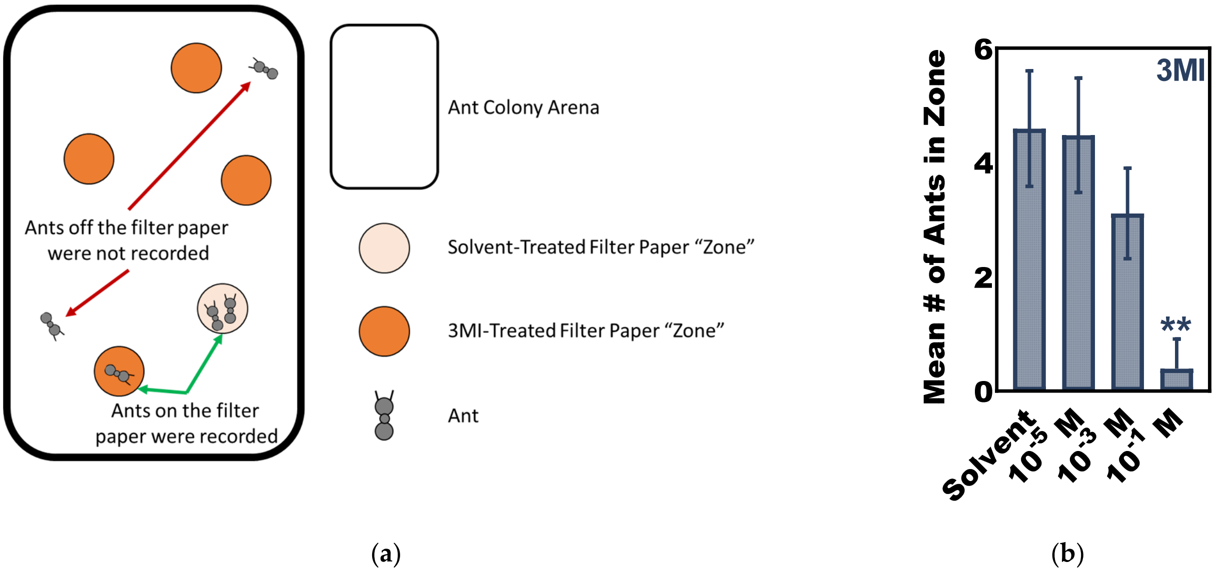

Figure S1. Colony-level valence in response to 3MI. (**a**) A schematic of the colony-level valence bioassay. Colonies were comprised of approximately 2,000-5,000 ants per colony. (**b**) Colony-level valence in response to solvent (DMSO) and serial dilutions of 3MI. One-Way ANOVA with Tukey's correction for multiple comparisons, P=0.0038 (**), N = 7.

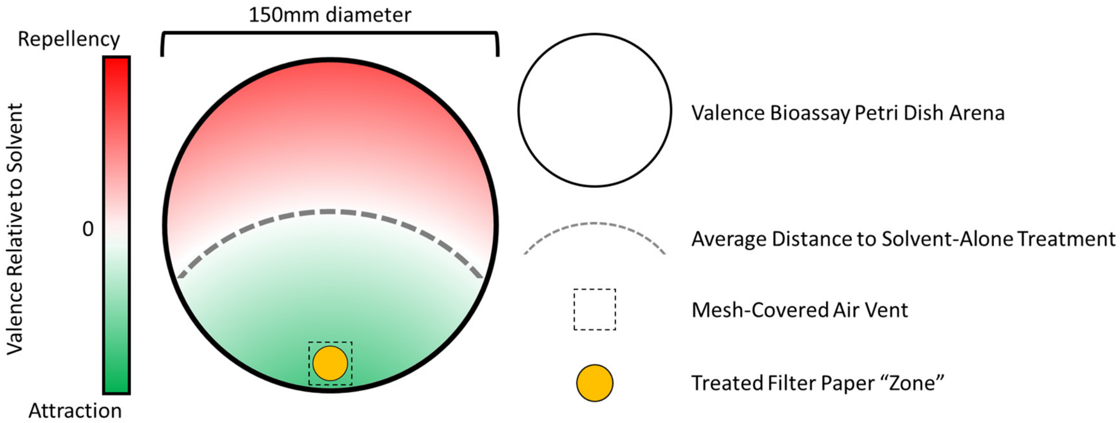

Figure S2. Valence bioassay for individual ants in response to 3MI. A schematic of the valence bioassay designed for assessing the responses of individual ants.

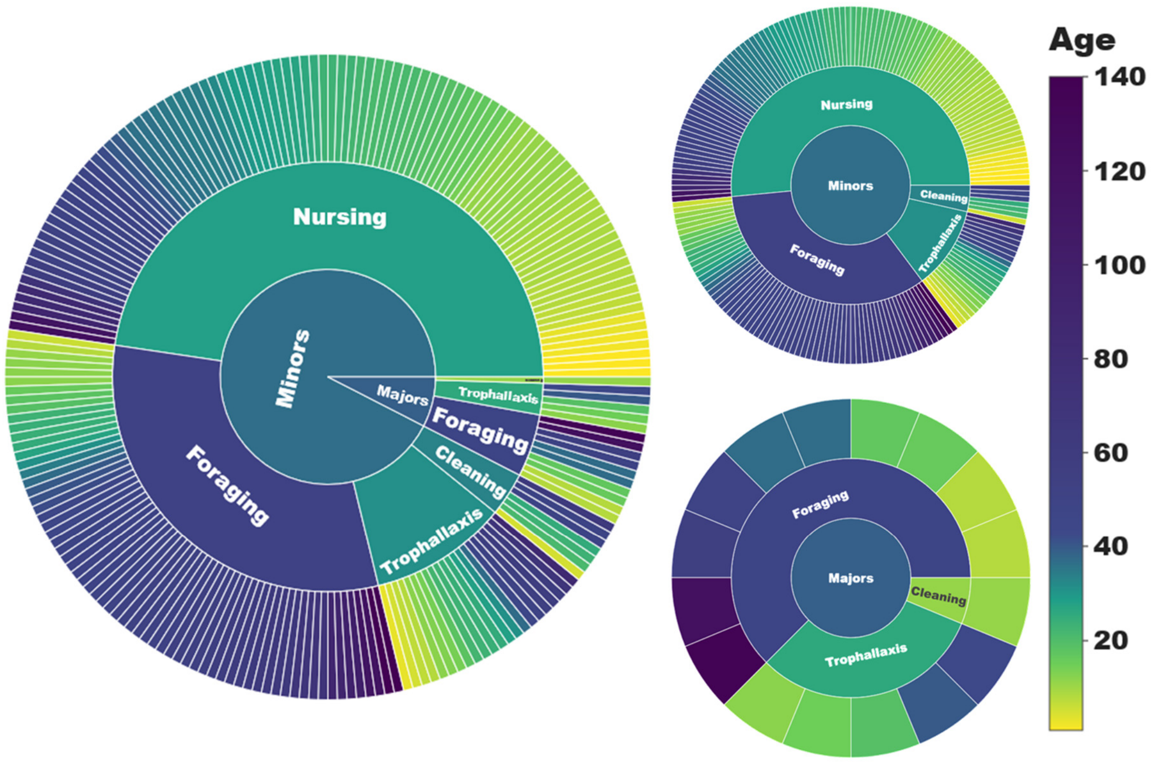

Figure S3. Caste-specific division of labor among *C. floridanus* workers. Sunburst charts with individual behavioral observation represented as leaves and broader categorizations (task group, worker caste) organized as roots. The color of the leaves corresponds to the actual age of the individual whereas the color of the roots corresponds to the mean age for that group. Minors and majors have a different repertoire of social behaviors. Minors were more active and exhibited an age-associated transition from nursing, trophallaxis, cleaning, and foraging whereas majors never nursed and seldom engaged in other tasks. N=196 minor workers observed performing social behaviors; N=16 major workers.

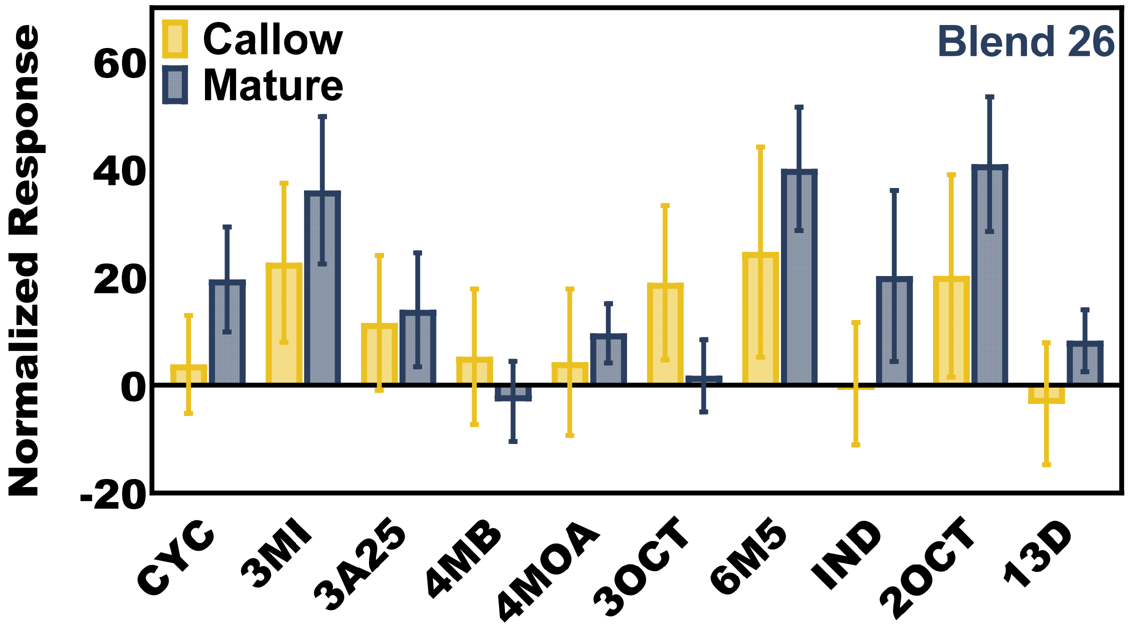

Figure S4. Responses of callow and mature minor workers to the unitary compounds present in ketone/indole Blend 26. A bar graph of the mean solvent (ND96) normalized EAG responses of callow minors (yellow) and mature minors (blue) to the unitary compounds found in odor Blend 26: 1,3-diphenylacetone (13D), 2-octanone (2OCT), 3-acetyl-2,5-dimethylfuran (3A25), 3-methylindole (3MI), 3-octanone (3OCT), 4-(4-methoxyphenyl)-2-butanone (4MB), 4'-methyoxyacetophenone (4MOA), 6-methyl-5-hepten-2-one (6M5), cyclohexanone (CYC), and indole (IND). N=7 callow minors; N=8 mature minors.

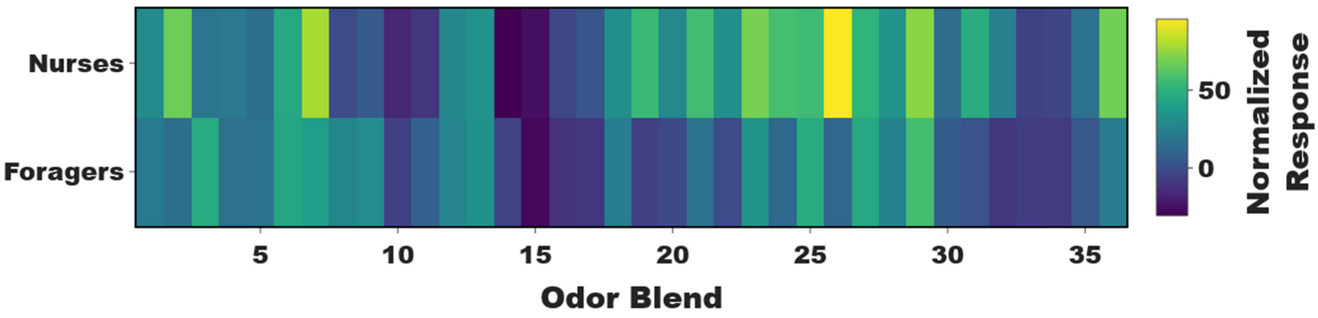

Figure S5. Responses of age-unknown nurses and foragers to 36 odor blends. A heatmap showing the mean solvent (ND96) normalized EAG responses of age-unknown minor nurses and minor foragers.

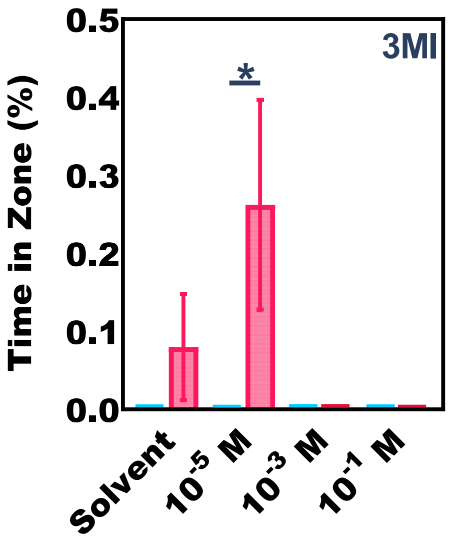

Figure S6. Foragers are significantly more attracted to 3MI at low concentrations compared to nurses. Mean percentage of time individual nurses (blue) and foragers (red) spent standing directly on top of the Whatman paper "zone" containing either solvent or serial dilutions of 3MI. Two-Way ANOVA with Tukey's correction for multiple comparisons, P=0.0154 (*), N=5 solvent, 10^{-3} M, 10^{-1} M; N=8 10^{-5} M.
